# Role of stem cell derivatives in inflammatory diseases

**DOI:** 10.3389/fimmu.2023.1153901

**Published:** 2023-03-14

**Authors:** Yuxi Yang, Yiqiu Peng, Yingying Li, Tingjuan Shi, Yingyi Luan, Chenghong Yin

**Affiliations:** ^1^ Department of Internal Medicine, Beijing Obstetrics and Gynecology Hospital, Capital Medical University, Beijing Maternal and Child Health Care Hospital, Beijing, China; ^2^ Department of Central Laboratory, Beijing Obstetrics and Gynecology Hospital, Capital Medical University, Beijing Maternal and Child Health Care Hospital, Beijing, China

**Keywords:** stem cells, exosome, treatment, inflammatory diseases, diagnose

## Abstract

Mesenchymal stem cells (MSCs) are pluripotent stem cells of mesodermal origin with the ability of self-renewal and multidirectional differentiation, which have all the common characteristics of stem cells and the ability to differentiate into adipocytes, osteoblasts, neuron-like cells and other cells. Stem cell derivatives are extracellular vesicles(EVs) released from mesenchymal stem cells that are involved in the process of body’s immune response, antigen presentation, cell differentiation, and anti-inflammatory. EVs are further divided into ectosomes and exosomes are widely used in degenerative diseases, cancer, and inflammatory diseases due to their parental cell characteristics. However, most diseases are closely related to inflammation, and exosomes can mitigate the damage caused by inflammation in terms of suppressing the inflammatory response, anti-apoptosis and promoting tissue repair. Stem cell-derived exosomes have become an emerging modality for cell-free therapy because of their high safety and ease of preservation and transportation through intercellular communication. In this review, we highlight the characteristics and functions of MSCs-derived exosomes and discuss the regulatory mechanisms of MSCs-derived exosomes in inflammatory diseases and their potential applications in clinical diagnosis and therapy.

## 1 Introduction

Mesenchymal stem cells (MSCs) are a class of non-hematopoietic multifunctional stem cells that originate from the mesoderm and were first discovered in the bone marrow by Friedenstein in the late 1960’s. They grow clonally against the wall and have a morphology similar to that of fibroblasts, with the potential for self-renewal and multidirectional differentiation into adipocytes, osteoblasts and chondrocytes *in vitro (* 1). Bone marrow and subcutaneous fat are common sources of cells (2). In addition, MSCs can also be isolated and prepared from muscle, lung, liver, pancreas, as well as from amniotic fluid and umbilical cord blood (3). In addition to having proliferative and self-renewal properties, the International Committee determined that MSCs must meet the following criteria: maintain adherent growth under standard laboratory conditions; express the typical MSC markers: CD73, CD90 and CD105, but not the haematopoietic markers: CD14, CD34 and CD45 or CD11b, CD19 and CD79α (4). MSCs play an important role in the normal development and maturation of hematopoietic stem cells (5) and are also involved in angiogenesis (6), production of various growth factors and cytokines to protect damaged or dead cells (7). In addition to their direct effects, MSCs can also release extracellular vesicles (EVs) to alter the microenvironment. EVs are a family of fluid-containing particles/vesicles which divided into three main categories: categories, microvesicles and exosomes (8).MSCs protect and regenerate damaged cells and attenuate immune responses by EVs delivery of a variety of proteins, including chemokines, growth factors and cytokines, membrane receptors, lipids, and various nucleic acids (9).

Exosomes are spherical vesicles composed of a double layer of lipid membranes, 40-160 nm in diameter, and are the most studied EVs type at present (10). Most cells in the body can secrete exosome, such as MSCs, *natural killer* and T lymphocyte cells, and neutrophils, which have many of the distinctive features of their parent cells, but also have their own unique characteristics, containing biologically active macromolecules such as DNA, RNA, nucleic acids, proteins and lipids, and regulates the function of receptor cells by transferring these substances to other receptor cells (11).

Inflammation, which can be classified as infectious inflammation caused by infection or non-infectious inflammation caused by factors such as tumors, trauma and autoimmune reactions. Inflammatory diseases are caused by inflammation, including infectious diseases, immune diseases, and destructive diseases. Previous studies have shown that inflammatory diseases involve all organs of the body, and inflammatory diseases not only lead to local apoptosis and necrosis but also deposit large amounts of extracellular matrix in the tissue, which is extremely destructive to the organism (12). Although inflammatory mediators are thought to be major players in triggering the inflammatory response and intercellular communication, a growing body of evidence supports the relationship between exosomes and inflammation (13). Exosomes play an important role in the pathogenesis and treatment of different inflammatory diseases by promoting proliferation, inhibiting apoptosis, reducing oxidative stress, and promoting cell repair and regeneration (14–16). However, exosomes also has challenges as a treatment in terms of tissue origin differences, age dependence, storage and transport, precise dosing and route of administration (17). There is still a long way to go before Exosomes are widely used as generic drugs for the treatment of inflammatory diseases

In this review, we provide a systematic review of the characteristics and effect of exosomes and the relationship between exosomes and inflammatory diseases; emphasized the current molecular and cellular mechanisms regarding the use of exosomes as a therapeutic approach for inflammatory diseases and its limitations and challenges.

## 2 Characteristics of stem cell derived exosomes

**Figure 1:**
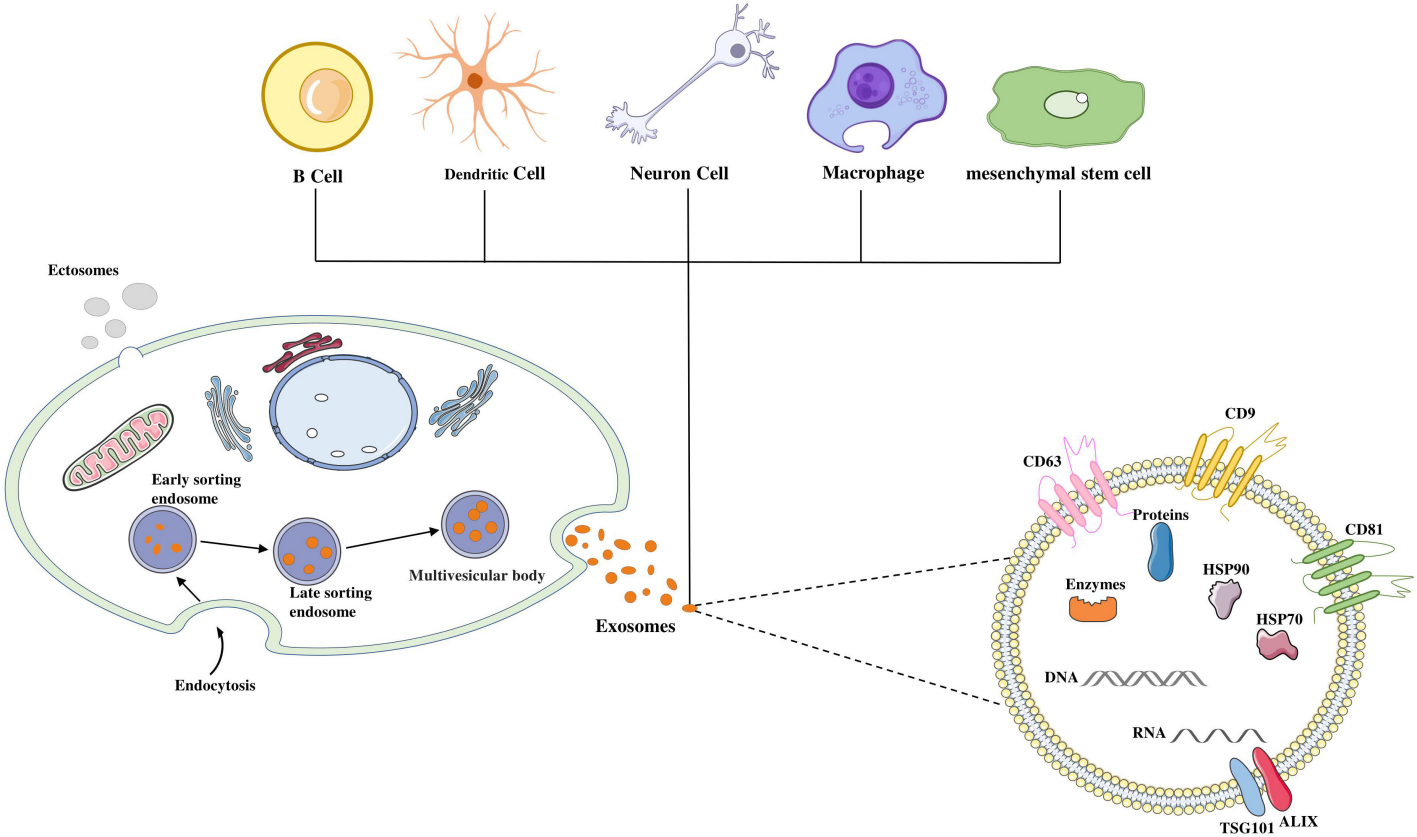
Biogenesis and composition of exosomes. Exosomes secreted by almost all types of cells in the body, such as MSCs, dendritic and B lymphocyte cells. Fluid and extracellular components can enter cells along with cell surface proteins through endocytosis and plasma membrane invagination. This process leads to the formation of early-sorting endosomes (ESEs), endoplasmic reticulum and pre-formed Golgi apparatus, develops into late endosomes (LSEs), and interconnects with cell membrane network structures to form intraluminal vesicles (ILVs) containing vesicle structures. the ILVs are ultimately secreted as exosomes of 40 ~ 160 nm in diameter through MVB fusion to the plasma membrane and cytosolic exocytosis. Exosomes expres EV surface markers CD63, CD9 and CD81, and also contain DNA, RNA and some common proteins, including MVB biogenesis proteins (Alix, TSG101, and ESCRT Complex), membrane transporter and heat shock proteins (HSP60, 70, and 90), lipid related proteins, and phospholipases.

Bonucci and Anderson first observed chondrocytes secreting small, secretory vesicles of approximately 100 nm in the late 1960s (18, 19). In 1983, scholars first discovered the existence of exosomes in the reticulocytes of sheep (20), which were initially thought to be a kind of excretion in the human body without any role and were not much concerned. Later, it was found to be functional, mainly involved in the exchange of information between substances and signal transduction, and was subsequently named exosomes in 1987 (21, 22). Exosomes are released by almost all cells except mature red blood cells (**Figure 1**).

Exosomes originate from endosomes, the process of exosome production involves plasma membrane invagination and subsequent formation of early-sorting endosomes (ESEs), which further mature into late-sorting endosomes (LSEs) and eventually form intracellular multivesicular bodies (MVBs) containing luminal vesicles (ILVs). When MVBs fuse with the plasma membrane, exosomes with diameters of 40 ~ 160 nm are released (**Figure 1**) (23).

Exosomes express the MSC surface markers CD73, CD44 and CD90 in addition to the EV surface markers CD9, CD63 and CD81 (24). They contain biological macromolecules such as membrane proteins, nuclear proteins, cytoplasmic proteins, nucleic acids, metabolites, DNA, mRNA and non-coding RNA. The mRNAs and miRNAs encapsulated in Exosomes form the molecular basis of its function. Common proteins in exosomes also play an important role, including membrane transporter and fusion proteins (GTPases, RAB protein and annexins), heat shock proteins (HSP60, 70, and 90), as well as other proteins that are also used as markers for exosomes (Alix, TSG101, and ESCRT Complex), phospholipases and lipid related proteins. Delivery of exosome contents can bind to target cells through three pathways: ligand-receptor binding, endocytosis and direct binding (25). Therefore, intercellular communication of exosomes in these three ways, thereby altering the function and activity of target cells (26).

Because exosomes contain growth factors, RNA, DNA and other components from parental cells, and transferred to the recipient cell *via* intercellular communication, they are widely used in the treatment of inflammatory diseases **Table 1** such as chronic inflammatory diseases, autoimmune diseases and destructive diseases and other inflammatory diseases (39). Exosomes act as intercellular messengers, carrying proteins and DNA, transporting RNA, and playing an important role in intercellular material and information transduction. For example, MSC-Exo significantly reduced pulmonary edema and lung protein permeability by reducing inflammatory cell invasion and inflammatory factor production in a mouse model of endotoxin-induced acute lung injury (40). Based on the functional similarity to parental cells, exosomes can cross the blood-brain barrier and are an effective cell-free reagent that may be safer in clinical treatment and may have fewer side effects compared to cell transplantation. However, its safety specification and methodological standardization have not yet been agreed upon in clinical studies. Therefore, these safety issues need to be further explored in future large-scale replications in clinical trials.

**Table 1:** The role of exosomes in the treatment of inflammatory diseases, as discussed in the text.

Disease types	Exosomes origin	Target	Effect	Role of exosome	Reference
Liver fibrosis	Human umbilical cord blood mesenchymal stem cell	Transcription factor Foxg1	Promoted rat liver regeneration and ameliorated liver injury after partial hepatectomy	Enhanced liver regeneration	(27)
Liver fibrosis	Kupffer cells	NADK mRNA	Inhibits fibrogenesis in hepatic stellate cells, inflammation in RHMs, and *de novo* lipogenesis in hepatocytes	Therapeutic	(28)
Inflammatory bowel disease	Mesenchymal stromal cells	Colonic macrophages	Downregulated inflammatory responses, maintained intestinal barrier integrity, and polarized M2b macrophages	Anti-inflammatory	(29)
Inflammatory bowel disease	Serum	Macrophages	Involved in the complement and coagulation cascade, which has been implicated in macrophage activation	Immunomodulatory and pro-inflammatory	(30)
Acute lung injury	Adipose-derived mesenchymal stromal cells	Macrophages	suppressed macrophage aggregation in lung tissues and inhibited IL-27 secretion, reduced pulmonary edema and pulmonary vascular leakage	Anti-inflammatory	(31)
Acute lung injury	Bone marrow mesenchymal stem cell	Macrophages	Alleviating lung pathological changes and pulmonary vascular permeability and attenuating inflammatory response	Relieving autophagy disorder	(32)
Atherosclerosis	Endothelial progenitor cells	Endothelial cells	Reduced the production of diabetic atherosclerotic plaques and inflammatory factors	Anti-inflammatory	(33)
Atherosclerosis	Bone marrow-derived mesenchymal stem cells	Mitogen-activated protein 4 kinase 4	Decreased inflammatory reaction, blood lipid, plaque area, MMP-9 expression and increased α-SMA expression, as well as inhibited apoptosis index	Anti-inflammatory	(34)
Rheumatoid arthritis	Human umbilical cord mesenchymal stem cells	T lymphocytes	Inhibited the proliferation and promoted apoptosis in T lymphocytes, downregulated RORγt mRNA and protein expression, decreased Th17 cell ratio, upregulated Foxp3 mRNA and protein expression, and increased Treg cell ratio in the spleen	Immunomodulatory	(35)
Rheumatoid arthritis	Synovial fibroblasts	FOXP3	Induce Th17 differentiation and inhibit Treg differentiation, with the increase of pro-inflammatory cytokines and decrease of anti-inflammatory cytokines.	pro-inflammatory	(36)
Multiple sclerosis	Human mesenchymal stem cells	microglia	Decreased neuroinflammation and upregulated the number of CD4+CD25+FOXP3+ regulatory T cells	Anti-inflammatory	(37)
Multiple sclerosis	Mesenchymal stromal cells	Oligodendrocyte progenitor cells and microglia	Promoted remyelination, increased polarization of M2 phenotype and blocked TLR2 signaling	Anti-inflammatory	(38)

## 3 Applications exosomes in inflammatory diseases

### 3.1 Exosomes and liver fibrosis

Fibrosis can occur in a variety of organs, and the main pathological changes are an increase in fibrous connective tissue and a decrease in parenchymal cells in the organ tissues, which can lead to structural destruction and functional decompensation of organs and even failure, posing a serious threat to human health and life. The liver is a central organ for metabolic and immune regulation, and most chronic liver diseases, such as fatty liver, viral hepatitis and autoimmune diseases can all lead to liver fibrosis. Liver fibrosis is an imbalance between the synthesis and degradation of the liver’s extracellular matrix (such as collagen glycoproteins and proteoglycans) that progresses further into liver cirrhosis (41). If left unprevented and untreated, liver fibrosis may eventually lead to cirrhosis, liver cancer and even liver failure.

Exosomes alleviate liver fibrosis by reducing inflammation and promoting liver tissue repair (42). Li et al. (43) showed that human umbilical cord MSCs-derived exosomes attenuated carbon tetrachloride (CCl4)-induced liver fibrosis in mice by restoring serum AST levels and reducing the deposition of cross-linked type I and type III collagen and decrease transforming growth factor (TGF)-β1 and phosphorylation Smad2in the liver. Through TGF-β1 production, Kupffer cells induce enhanced expression of pro-fibrotic genes (waveform protein, type I collagen, fibronectin and α-SMA) in hematopoietic stem cells, leading to the development of liver fibrosis. Exosomes can reduce liver fibrosis by inhibiting the production of inflammatory factors such as interleukin-6 (IL-6), interleukin-1β (IL-1β) and tumor necrosis factor-α (TNF-α) and pro-fibrotic transforming growth factor-β1 in Kupffer cells (44). Based on these previous studies, Qu and colleagues designed miRNA-181-5p overexpression of adipose tissue-derived mesenchymal stem cells (MSCsmiRNA-181-5p-Exos) to increase autophagy levels in liver fibrosis by promoting the expression of autophagy-related protein Beclin-1 and inhibiting the expression of anti-apoptotic protein Bcl-2. It also significantly downregulated the expression of pro-fibrotic genes (waveform protein, type I collagen, fibronectin and α-SMA) in hematopoietic stem cells, thereby attenuating CCl4-induced liver fibrosis in mice (45).

Exosomes also contain miRNAs, such as miRNA-181-5, MiR-690, MiR-223 and miR-122, which have antifibrotic effects in liver fibrosis. Recent studies have indicated that exosomal miR-124 attenuates liver injury after partial hepatectomy in rats and promotes hepatocyte proliferation and liver regeneration by inhibiting Foxg1 expression (27). Similarly, MiR-690 treatment has been found to reduce liver fibrosis and steatosis and restore specific Kupffer cell functions (28). Hou et al. (46) demonstrated that IL-6 treatment promotes the release of miR-223-enriched exosomes from macrophages and thus exerts an anti-fibrotic effect in Nonalcoholic fatty liver disease patients.

### 3.2 Exosomes and inflammatory bowel disease

Inflammatory bowel disease (IBD) is a non-specific inflammatory disease of the intestine involving the ileum, rectum and colon mediated by abnormal immunity and infection. Histologically, IBD is characterized by shortening and branching of the crypt, crypt abscesses or cryptitis. IBD includes Crohn’s disease (CD) and ulcerative colitis (UC), characterized by disruption of the intestinal mucosal barrier, imbalance of immune cells and their secreted cytokines, and dysregulation of the immune response to intestinal microorganisms, causing chronic intestinal mucosal damage (47).

Macrophages are important in maintaining intestinal homeostasis, they are involved in the regulation of BD pathogenesis, and some evidence supports the ability of exosomes to activate macrophages (48). Bone marrow mesenchymal stem cells-derived exosomes for the treatment IBD leads to polarization of M2b macrophages, reduces inflammation and maintenance of the integrity of the intestinal barrier.

These exosomes are enriched with proteins involved in the regulation of biological processes in anti-colitis, in particular metallothionein-2, which is necessary for suppression of inflammatory responses thereby attenuating colitis (29). Wong et al. identified the role of circulating exosomal proteins in bone marrow-derived macrophages(BMDMs) activation by establishing a model of acute colitis in athymic mice. Proteomics and bioinformatics analysis of RAW264.7 macrophage-treated sera identified 56 proteins (include immunoglobulins and acute phase proteins) that are involved in coagulation and complement cascades and ultimately activate macrophages (30). Further, some exosomal heat shock proteins such as HSP72, HSP20 and HSP90 have also been shown to be associated with the development of IBD (49).

In addition to proteins, exosome-mediated miRNAs play an important role in IBD. Exosomal miRNAs coordinate the immune system by regulating the functions of T cells, dendritic cells (DCs), macrophages and NF-κB-related signaling pathways. Such as miR-146a and miR-155, can regulate the inflammatory response in IBD. miR-155 deficiency inhibits the function and growth of DC in intestinal lamina propria and thereby ameliorates intestinal inflammation (50). miR-1246 also activates T cells and thus pro-inflammatory nuclear factors in active IBD (51). A recent study found that platelet-derived exosomal MiR-223 regulates immune cell and intestinal barrier function in IBD by blocking the MAPK pathway (downregulating phosphorylation ofc-JNK, ERK and p38) and NF-κB p65 nuclear translocation and inhibiting intercellular adhesion molecule-1 in inflammation (52). Similarly, it was shown that after treatment with exosomes, microbiome alterations in colitis model mice return to concordance compared to healthy mice. Exosome treatment also improved overall intestinal health and healing by increasing the number of Lgr5+ intestinal stem cells, promote intestinal angiogenesis and stimulating intestinal epithelial cell proliferation (53).

### 3.3 Exosomes and acute lung injury

Acute lung injury (ALI) is an injury to alveolar epithelial cells and capillary endothelial cells caused by various direct and indirect injury factors, resulting in diffuse interstitial lung and alveolar edema, which can eventually progress to pulmonary fibrosis and therefore has a high clinical mortality rate (54). Recently, an increasing number of reports have showed that exosomes play an important role in tissue repair, immune response and reduction of pro-inflammatory cytokines in sepsis and non-sepsis-induced ALI (55–57). Li et al. (58) found that injection of MSCs-derived exosomes into an ALI mouse model significantly reduced neutrophil and macrophage inflammatory protein-2 levels. Moreover, alveolar macrophage-derived exosomes inhibit secretion of pro-inflammatory factors IL-1β and TNF-α and increased secretion of anti-inflammatory factors TGF-β (59). Similarly, Wang et al. found that MSCs-derived exosomes could alleviate sepsis-induced lung injury by inhibiting the release of IL-27, suppressing the expression of pro-inflammatory factors IL-6, IL-1β and TNF-α, and inhibiting the aggregation of pulmonary macrophages (31). Exosomes not only play an anti-inflammatory role, but also regulate apoptosis. For example, Sun et al. reported that exosomes regulate the expression of mtDNA damage makers and apoptosis-related proteins through the nod-like receptor protein 3 (NLRP3) pathway to alleviate mitochondrial DNA damage and alveolar epithelial cell apoptosis and mitigate lung injury (60). Adipose stem cell-derived exosomes attenuate sepsis-induced lung injury by promoting autophagy, reducing alveolar epithelial cell apoptosis and pro-inflammatory factor expression (61). Exosomes promote the regeneration of alveolar epithelial and endothelial cells and attenuate lung injury. Dinh et al. found that exosomes of pulmonary spherocyte origin attenuate fibrosis by reconstructing normal alveolar structure, suggesting the therapeutic potential of exosomes by increasing cell regeneration (62). Wu et al. also reported that exosomes of endothelial progenitor cell origin improve endothelial cell function by promoting the migration and proliferation of endothelial cells to attenuate lipopolysaccharide (LPS) induced inflammation in the lung (63).

Exosomes act as carriers of miRNAs that regulate pathological processes in lung diseases. For example, MSCs-derived exosomes inhibit NF-κB and hedgehog signaling pathways by disrupting IKKβand silencing Ikbkb, thus delivering miR-182-5p and miR-23a-3p and reversing the progression of LPS-induced lung injury and fibrosis (64). Exosomal miR-1-3p promotes apoptosis and cytoskeleton contraction, inhibits cell proliferation and impairs vascular barrier function involved in ALI progress. MiR-1-3p may be a potential therapeutic target for sepsis-induced ALI (65). Liu et al. found that miR-384-5p was enriched in bone marrow mesenchymal stem cell-derived exosomes and alleviated impaired autophagy in alveolar macrophages in an LPS-induced ALI model by targeting Beclin-1 (32).Besides, MiR-377-3p released from MSCs-derived exosomes attenuated lipopolysaccharide-induced ALI by targeting the regulatory-associated protein of mTOR to induce autophagy (66). Exosomes can also regulate ALI in other ways. Exosomes from adipose-derived MSCs can mitigate ALI by transferring mitochondrial components to improve oxidative phosphorylation levels and macrophage mitochondrial integrity, thereby restoring airway macrophage immune and metabolic homeostasis (67).

### 3.4 Exosomes and Atherosclerosis

Atherosclerosis is an inflammatory disease of the blood vessels, and impaired lipid metabolism is the basis of atherosclerosis. It is characterized by lesions of the affected arteries starting from the intima, generally with accumulation of lipids and complex sugars, hemorrhage and thrombosis, followed by proliferation of fibrous tissue and calcium deposits, and gradual metamorphosis and calcification of the middle layer of the arteries, resulting in thickening and hardening of the arterial walls and narrowing of the lumen and activation of inflammatory pathways. Atherosclerosis is the leading cause of cardiovascular disease and the leading cause of death worldwide.

Atherosclerosis is caused by a combination of multiple factors, and the pathogenesis is complex and has not been fully elucidated. The main risk factors are hypertension, hyperlipidemia and smoking, as well as diabetes, obesity and genetic factors (68, 69). Lipid infiltration, endothelial cell damage theory, vascular smooth muscle proliferation, inflammation, macrophage polarization and so on are involved in the process of atherosclerosis (70). Therefore, intercellular communication is important in atherosclerosis, and exosomes are also participants in intercellular communication. Extracellular vesicles as well as exosomes are involved in atherosclerotic microcalcifications (71). Exosomes can be used not only as biomarkers for the diagnosis of atherosclerosis, but also as potential therapeutic targets for atherosclerosis (10, 72).

Macrophages differentiated from monocytes play an important role in atherosclerosis, and macrophage-derived exosomes are involved in the whole process of atherosclerosis (73). It has been shown that atherosclerosis-stimulated treated mouse macrophages are enriched in miR-146a, which reduces cell migration and promotes macrophage retention in the vessel wall thereby exacerbating atherosclerosis. Suggesting a biomarker role for miR-146a in atherosclerosis progress (74). Similarly, the exosome miR-146 from atherogenic macrophages was found to promote the development of atherosclerosis by promoting neutrophil extracellular traps (75). Furthermore, the BMDMs-derived exosome miR-21-3p promoted the proliferation and migration of vascular smooth muscle cells(VSMCs) thereby promoting atherosclerosis (76). In addition to macrophages, VSMCs-derived exosomes are also key regulators of atherosclerosis. For example, exosomes from VSMCs were found to accelerate calcification and thus promote atherosclerosis by propagating precalcification signals (77). Besides, exosomes are released by VSMCs and deposit them in pre-calcified vessels, which may provide the conditions for vessel wall calcification (78).

Although exosomes can promote atherosclerosis, on the other hand, exosomes can also reduce the extent of atherosclerosis. For example, exosomal miRNAs play an important role in the suppression of atherosclerosis. Bai et al. showed that endothelial progenitor cell-derived exosomes significantly reduced the production of atherosclerotic plaque and inflammatory factors and improved atherosclerotic diabetes by improving endothelial dysfunction (33). In addition, in high-fat diet-fed apolipoprotein E-deficient (ApoE(-/-)) mice, MSCs-derived exosomes reduced the size of atherosclerotic plaques and macrophage infiltration in plaques and promoted the polarization of M2 macrophages (79). Similarly, the mouse bone marrow MSCs-derived exosome miR-512-3p prevents atherosclerosis by reducing apoptosis and inflammatory factor expression to inhibit endothelial cell dysfunction (80). Lin et al. demonstrated that exosomal miR-125b-5p from bone marrow mesenchymal stem cells can be used as a treatment for atherosclerosis by reducing plaque size, lipids, and inhibiting apoptosis in mice with atherosclerosis (34). In a high-fat diet-fed ApoE(-/-) mouse model, BMDMs-derived exosomes were able to reduce inflammation and hematopoiesis while inducing a macrophage polarization. BMDMs-derived exosomes containing miR-146b, miR-99 and miR-378a have anti-inflammatory properties, inhibit inflammation and accelerate M2 polarization in BMDMs thereby inhibiting atherosclerosis (81).

### 3.5 Exosomes and rheumatoid arthritis

Rheumatoid arthritis (RA) is a chronic, predominantly inflammatory synovitis systemic disease of unknown etiology. It is characterized by polyarticular, symmetric, aggressive joint inflammation of the small joints of the hands and feet and also damage to extra-articular organs, including the heart, lungs, kidneys, eyes, skin and nervous system (82). MSCs-derived exosomes have been shown to be beneficial in treating RA by reducing bone destruction and erosion, joint inflammation and mitigating the formation of panniculitis through immunomodulation, anti-inflammation and differentiation (83, 84). RA is primarily due to an imbalance between effector T cell subpopulations of T helper type 1/T helper type 17 (Th1/Th17) and IL-10 producing immunomodulatory Treg, which leads to injury and inflammation in target tissues. Since immunomodulation by bone marrow MSC-derived exosomes is most effective against CD4 cells, it is not surprising that exosomes treat autoimmune diseases (85). For example, Ma et al. found that both human umbilical cord-MSCs-derived exosomes inhibited T cell proliferation, promoted T cell apoptosis, decreased rorr γ levels, increased forkhead box P3(Foxp3) levels, and regulated Treg/Th17 cell homeostasis, thereby inhibiting synovial proliferation and delaying the progression of RA through *in vitro* and *in vivo* experiments (35). Dendritic cells treated with IL-10 have been found to release exosomes and reduce the severity of RA (86). In addition, dc exosomes treated with IL-10 prevented the invasion of RA in mice and reduced the severity of existing arthritis (87).

In addition, exosomal miRNAs play an important role in mitigating the development of RA. Studies in RA model mice showed that increased expression of exosomal miR-424 targeting Foxp3 significantly inhibited T cell differentiation, decreased Treg cells and increased Th17 cells. Thus, exosomal miR-424 overexpression may mitigate RA progression (36). Exosomal miR-204-5p can translocate to synovial fibroblasts and inhibit cell proliferation. Wu et al. found that exosomal miR-204-5p expression was negatively correlated with disease parameters such as rheumatoid factor, erythrocyte sedimentation rate, and c-reactive protein in RA patients and could be a potential biomarker target for RA therapy (88). Similarly, MSCs-derived exosomal miR-192-5p significantly reduced the levels of pro-inflammatory cytokines, including PGE2, IL-1β and TNF-α, in synovial tissue and serum of collagen-induced arthritis (CIA) rats, and decreased the release of NO and inducible NO synthase (iNOS) in serum (89). A recent study showed that miR-146a-transduced MSCs-derived exosomes also increased Foxp3, TGFβ and IL-10 gene expression in CIA mice, and that exosomes appear to promote direct intracellular transfer and immunomodulatory effects of intercellular MiRNA and represent a possible therapeutic strategy for RA (90). Lim et al. also found that serum exosomal miR-1915-3p levels were negatively correlated with serum c-reactive protein levels, predicting that miR-1915-3p could serve as a potential marker of RA disease activity (91). By preparing human mesenchymal stem cell exosomes overexpressing miRNA-124a, Meng et al. found that this exosome pretreatment inhibited the migration and proliferation of fibroblast-like synoviocytes during coincubation and promoted apoptosis of the cell, which may be a suitable vehicle for therapeutic drugs (92).

### 3.6 Exosomes and multiple sclerosis

Multiple sclerosis (MS) is an autoimmune disease characterized by inflammatory demyelinating lesions in the white matter of the central nervous system caused by autoimmune and viral infections. The most frequent sites of involvement are the periventricular white matter, optic nerve, spinal cord, brainstem and cerebellum (93). The characteristic pathological changes of MS are the invasion of autoreactive CD4+ T cells (especially th17 and Th1 cells) into the central nervous system (CNS) and multiple demyelinated plaques in the white matter of the CNS, mostly located around the lateral ventricles, with reactive gliosis and axonal damage (84). In addition to this, microRNAs are involved in the pathological changes of MS (94). However, current therapeutic approaches regarding MS are still limited, mostly immunomodulatory treatments (including immunomodulators and immunosuppressants, etc.) are performed. In addition to crossing the blood-brain barrier, exosomes contain a range of anti-inflammatory and neuroprotective agents that may have a protective effect on immune modulation and demyelinated tissue repair (95).

The understanding of exosomes in the diagnosis and treatment of MS has progressed significantly in recent years. Riazifar et al. found that MSCs-derived exosomes stimulated by IFN γ attenuated neuroinflammation and demyelination and upregulated the number of CD4 + CD25 + FOXP3 + regulatory T cells (Tregs) in a mouse model of experimental autoimmune encephalomyelitis (EAE) (37). It was also found that MSCs-derived exosomes treatment promoted myelin regeneration in microglia in the EAE mouse model, increased polarization of the M2 phenotype and blocked TLR2 signaling, providing the animal and cellular basis for this cell-free exosome treatment of MS (38). Not only that, exosomal miRNA can be used as a biomarker to diagnose MS. In a cohort study, five miRNA (hsa-miR-140-5p, hsa-miR-484, hsa-miR-486-5p, hsa-miR-320a and hsa-miR-320c) were significantly different between MS patients and healthy subjects (96). Similarly, another study identified 15 separate miRNAs that were differentially expressed in serum exosomes of patients with active versus quiescent MS disease after treatment (97).

## 4 Clinical applications of exosomes

**Figure 2:**
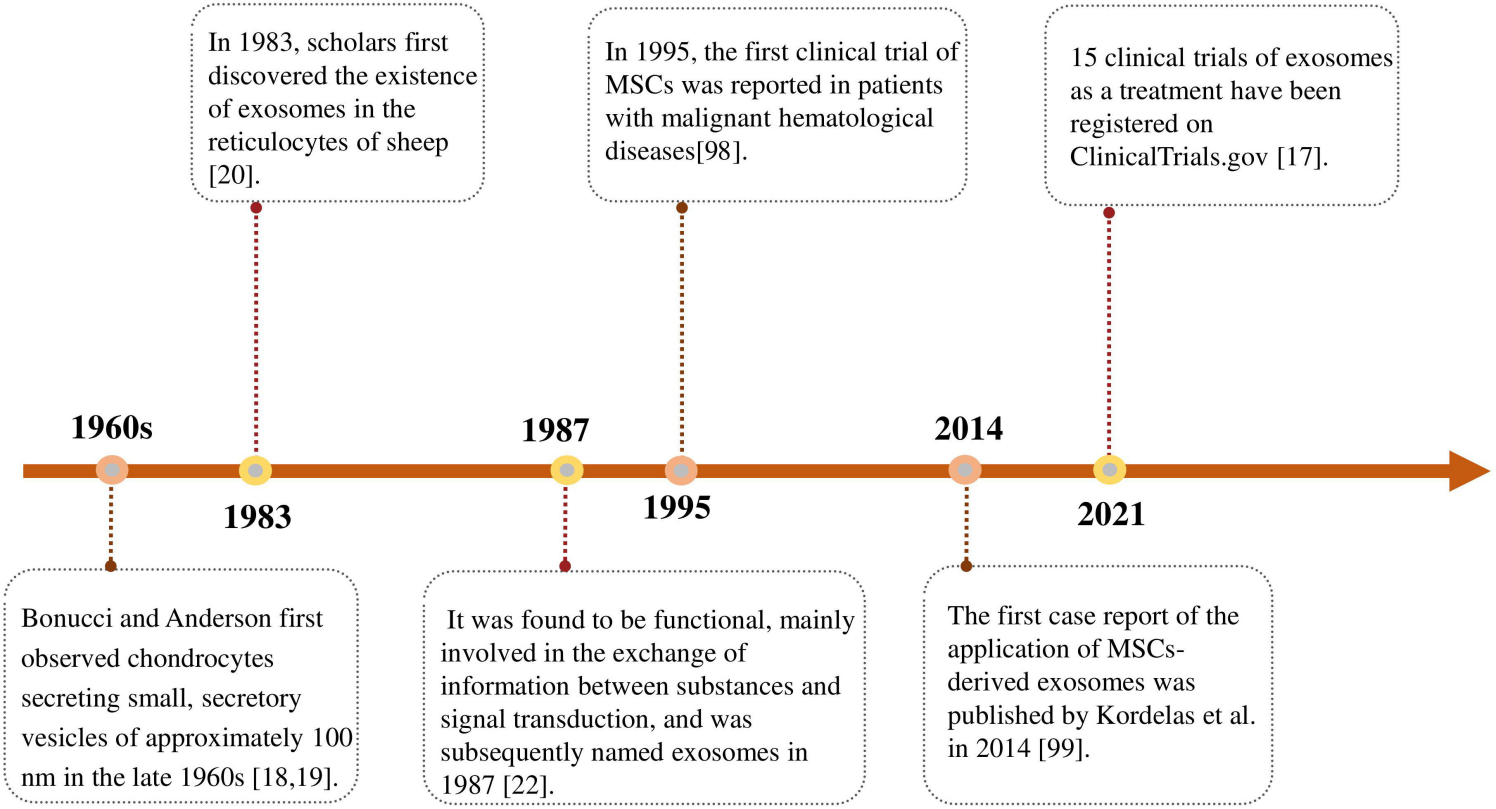
Timeline of advances in the research on exosomes. The first discovery and designation of exosomes and their clinical application as therapeutic tools.

In 1995, the first clinical trial of MSCs was reported in patients with malignant hematological diseases. Lazarus et al. (98) confirmed that no adverse infusion reactions were observed after patients were treated with MSCs (**Figure 2**). A growing number of clinical trials have investigated the effectiveness and safety of MSCs in the treatment of various diseases.

Exosomes are safer and more effective than MCSs treatment as a novel cell-free treatment strategy. Inspired by the promising results observed in preclinical studies on the therapeutic efficacy of MSCs-derived exosomes, the first case report of the application of MSCs-derived exosomes was published by Kordelas et al. in 2014. Four doses of exosomes were obtained from 4 × 10^7^ bone marrow-MSCs supernatant isolated and injected every 2 ~ 3 days for individualized treatment of patients with refractory graft-versus-host disease. After treatment, patients showed a significant reduction in pro-inflammatory cytokines and significant improvement in clinical symptoms (99).

MSCs-derived exosomes seem to be an interesting approach for clinical trials in various kind of diseases, especially those with an inflammatory component. The beneficial effects of MSCs-derived exosomes can be enhanced by genetic modifications and bioengineering, drug encapsulation or stimulation with different growth factors. As of December 2022, 295 clinical studies involving exosomes are listed at www.clinicaltrials.gov, with 98 trials (33.2%) in recruiting phase, 31 trials (10.5%) in not yet recruited phase, only 71 studies (24.1%) have been completed. Of these completed studies, most of which used exosomes as a diagnostic tool, those related to inflammatory diseases were shown in **Table 2**. To date, among the 295 trials using MSC-derived exosomes:46 trials in phase 1, 46 trials in phase 2, 73 trials combined phase 1/2; 6 trials in phase 3, 48 trials combined phase 2/3; 2 trials in phase 4 trials, and 78 trials unspecified phases.

**Table 2:** The role of Exosomes in the diagnose and treatment of inflammatory diseases, as discussed in the text.

Trial ID	Study title	Condition of disease	Source of exosome	Age	Trial Phase	First posted	study completion date
NCT04276987	A Pilot Clinical Study on Inhalation of Mesenchymal Stem Cells Exosomes Treating Severe Novel Coronavirus Pneumonia	Severe Novel Coronavirus Pneumonia	MSCs-derived exosome	18 Years to 75 Years (Adult, Older Adult)	Phase 1	February 19, 2020	July 31, 2020
NCT04849429	Intra-discal Injection of Platelet-rich Plasma (PRP) Enriched With Exosomes in Chronic Low Back Pain	Degenerative Disc Disease	Platelet-rich Plasma-derived exosome	18 Years to 60 Years (Adult)	Phase 1	April 19, 2021	July 25, 2022
NCT04491240	Evaluation of Safety and Efficiency of Method of Exosome Inhalation in SARS-CoV-2 Associated Pneumonia.	SARS-CoV-2 PNEUMONIACOVID-19	MSCs-derived exosome	18 Years to 65 Years (Adult, Older Adult)	Phase1, Phase 2	July 29, 2020	October 20, 2020
NCT01860118	LRRK2 and Other Novel Exosome Proteins in Parkinson’s Disease	Parkinson’s Disease	unspecified	21 Years and older (Adult, Older Adult)	Not Applicable	May 22, 2013	June 21, 2016
NCT03811600	Exosomes Implication in PD1-PD-L1 Activation in OSAS	Sleep Apnea Syndromes, Obstructive Cancer	Peripheral blood-derived exosome	18 Years and older (Adult, Older Adult)	Not Applicable	January 22, 2019	October 14, 2020
NCT04879810	Plant Exosomes +/- Curcumin to Abrogate Symptoms of Inflammatory Bowel Disease	Inflammatory Bowel Disease	plant cells-derived exosome	18 Years and older (Adult, Older Adult)	Not Applicable	May 10, 2021	August 2, 2022
NCT03202212	Effect of Mixed On-line Hemodiafiltration on Circulating Markers of Inflammation and Vascular Dysfunction	Chronic Kidney Failure, Dialysis Related Complication	plasmatic exosomes	18 Years and older (Adult, Older Adult)	Phase 1, Phase 2	June 28, 2017	November 11, 2014
NCT00331331	The Vitreous Proteome and Inflammatory Mediators in Ocular Inflammatory Disease	Uveitis, Vasculitis, Ocular and Inflammatory Disease	unspecified	18 Years and older (Adult, Older Adult)	Not Applicable	May 29, 2006	February 14, 2007
NCT03415984	Prevalence of Age Related Macular Degeneration (ARMD) in Parkinson’s Patients and Assesment of the Role of L-DOPA (AMD-PARK)	Parkinson Disease, Age Related Macular Degeneration	retinal pigmentary epithelium-derived exosome	50 Years and older (Adult, Older Adult)	Not Applicable	January 30, 2018	January 15, 2019
NCT04134676	Therapeutic Potential of Stem Cell Conditioned Medium on Chronic Ulcer Wounds	Chronic Ulcer	MSCs-derived exosome	18 Years to 80 Years (Adult, Older Adult)	Phase 1	October 22, 2019	June 10, 2020
NCT03984006	Early Detection of Autoimmune Thyroid Heart Disease *Via* Urinary Exosomal Proteins	Thyroid Diseases, Heart Failure	unspecified	20 Years to 80 Years (Adult, Older Adult)	Not Applicable	June 12, 2019	May 14, 2021
NCT04623671	Intravenous Infusion of CAP-1002 in Patients With COVID-19 (INSPIRE)	Covid19	Cardiosphere-Derived Cells(CDCs)-derived exosome	18 Years and older (Adult, Older Adult)	Phase 2	November 10, 2020	February 4, 2022
NCT04493242	Extracellular Vesicle Infusion Treatment for COVID-19 Associated ARDS (EXIT-COVID19)	Covid19, ARDS, Pneumonia and Viral	bone marrow MSCs-derived exosome	18 Years to 85 Years (Adult, Older Adult)	Phase 2	July 30, 2020	May 22, 2021
NCT02890134	Molecular Reclassification to Find Clinically Useful Biomarkers for Systemic Autoimmune Diseases: Inception Cohort (PRECISESADSI)	Systemic Autoimmune Diseases	plasma and urine derived-exosome	18 Years and older (Adult, Older Adult)	Not Applicable	September 7, 2016	July 2018
NCT01159288	Trial of a Vaccination With Tumor Antigen-loaded Dendritic Cell-derived Exosomes	Non Small Cell Lung Cancer	tumor antigen-loaded dendritic cells-derived exosomes	18 Years to 70 Years (Adult, Older Adult)	Phase 2	July 9, 2010	December 19, 2015

Despite more than 1,000 trials in more than 40 countries using various types of msc or MSC-free derivatives, only nine MSC-based products are approved globally for the treatment of regenerative or immune-related diseases (100). Therefore, further research is needed to establish therapeutic doses, suitable culture conditions and routes of administration of MSCs, and standardized protocols for exosome isolation and storage, so that exosomes can be widely used in the diagnosis and treatment of different diseases.

## 5 Perspectives on challenge in exosomes

Despite the advantages of MSCs therapy, there are still problems with cell senescence and differentiation, and clinical outcomes are not promising (17). MSCs also have a growth-promoting effect on different types of cancer. Moreover, there are some risks associated with cell treatment, such as immune rejection, viral infection, storage methods and transport issues (21). MSCs-derived exosomes represent a new, cell-free drug that effectively attenuates clear systemic inflammation. Both topical and systemic administration have the potential to effectively suppress deleterious immune responses in inflamed tissues by reducing apoptotic and pro-inflammatory cytokines, alleviating cellular dysfunction, and promoting the survival and regeneration of damaged parenchymal cells (101). MSCs exert a therapeutic effect by paracrine action, MSCs-derived exosomes can replace their parental cells and have greater advantages: they cannot self-replicate, avoiding to a large extent the risk of tumorigenicity (102); as nanoparticles, exosomes are safer than cell therapy, they are biocompatible and have low immunogenicity, allowing them to cross blood-brain barrier (103); exosomes are protected by their lipid membrane structure by internal biomolecular activity and can be stored at -80°C for a long time without easy inactivation (104); Exosomes act as natural carriers of exogenous nucleic acids and drugs and can be loaded in donor cells and then released into the extracellular environment (105). In addition, exosome can also prevent miRNAs from being degraded, allowing miRNAs to negatively regulate the expression of target proteins (39).

Although there have been many preclinical studies involving MSCs -derived exosomes, several important issues remain unresolved. First, when MSCs from different tissues are in different states of differentiation, exosomes assembly may differ in molecular content and type, with significant tissue origin and age-dependent differences in immunosuppressive and tissue regenerative capacity, thus affecting their function in recipient cells and thus therapeutic efficacy. Secondly, the miRNA of MSCs-derived exosomes is not randomized into exosomes, however, the sorting mechanism that adjusts and selects cells from the parent cells is not known (106). Finally, the beneficial effects of MSCs-derived exosomes depend mainly on mRNA, miRNAs, anti-apoptotic and immunosuppressive proteins, and further clinical trials should confirm the exact disease-specific MSCs source molecules. In addition, route of administration and the precise dose of MSCs-derived exosomes should be determined for different inflammatory disease to prevent uncontrolled immunosuppression of MSCs-derived exosomes receptors. Different laboratories use different methods to isolate and purify MSCs-derived exosomes, therefore, an efficient method for standardizing the mass production of MSCs -derived exosomes is essential (107).

Before exosomes can be used in large numbers as clinical therapeutic agents, considerable researches are also needed to identify those components of exosomes that are of diagnostic and therapeutic importance, particularly as key targets in the regulation of inflammatory disease processes.

## 6 Conclusions

Most of the therapeutic effects of MSCs depend on their paracrine action, and exosomes are particularly suitable for studying disease diagnosis or prognosis due to their parental cellular properties. Exosomes also act as membrane nanocarriers that modify the activity and function of target cells through intercellular communication thus playing a role in inflammation. The growing interest in exosomes and the accumulating findings suggest that their role in inflammatory diseases is extensive and complex. Therefore, research should be continued to elucidate the properties of exosomes and the whole process that mediates the inflammatory response. Understanding the role of the various exosomal contents and their respective functions will be key to understanding the diagnostic and therapeutic tools that exosomes play. In addition to this, once key questions surrounding the isolation, transport, dosing, storage, donor and tissue sources of MSCs-derived exosomes are answered, it will realize its full potential as a new therapy in treatment inflammatory diseases. Continued extensive researches are still needed in the future to gain a deeper understanding of exosomes in treatment and diagnosis of inflammatory diseases and to provide new clinical insights and solutions.

## Author contributions

YY design of the work and write the manuscript. YP, YLi and TS make the table of the manuscript; YLu and CY substantively revised the manuscript. All authors contributed to the article and approved the submitted version.
